# A Simple ERP Method for Quantitative Analysis of Cognitive Workload in Myoelectric Prosthesis Control and Human-Machine Interaction

**DOI:** 10.1371/journal.pone.0112091

**Published:** 2014-11-17

**Authors:** Sean Deeny, Caitlin Chicoine, Levi Hargrove, Todd Parrish, Arun Jayaraman

**Affiliations:** 1 Rehabilitation Institute of Chicago, Center for Bionic Medicine, Chicago, IL, United States of America; 2 Northwestern University, Feinberg School of Medicine, Chicago, IL, United States of America; ARC Centre of Excellence in Cognition and its Disorders (CCD), Australia

## Abstract

Common goals in the development of human-machine interface (HMI) technology are to reduce cognitive workload and increase function. However, objective and quantitative outcome measures assessing cognitive workload have not been standardized for HMI research. The present study examines the efficacy of a simple event-related potential (ERP) measure of cortical effort during myoelectric control of a virtual limb for use as an outcome tool. Participants trained and tested on two methods of control, direct control (DC) and pattern recognition control (PRC), while electroencephalographic (EEG) activity was recorded. Eighteen healthy participants with intact limbs were tested using DC and PRC under three conditions: passive viewing, easy, and hard. Novel auditory probes were presented at random intervals during testing, and significant task-difficulty effects were observed in the P200, P300, and a late positive potential (LPP), supporting the efficacy of ERPs as a cognitive workload measure in HMI tasks. LPP amplitude distinguished DC from PRC in the hard condition with higher amplitude in PRC, consistent with lower cognitive workload in PRC relative to DC for complex movements. Participants completed trials faster in the easy condition using DC relative to PRC, but completed trials more slowly using DC relative to PRC in the hard condition. The results provide promising support for ERPs as an outcome measure for cognitive workload in HMI research such as prosthetics, exoskeletons, and other assistive devices, and can be used to evaluate and guide new technologies for more intuitive HMI control.

## Introduction

The fields of rehabilitation and medical technology have seen significant recent advances that incorporate human-machine interaction (HMI), including the use of exoskeletons designed to enable ambulation in patients with spinal cord injury (SCI) and stroke [1], [2], robotic aids for surgery [3], and myoelectric control of prostheses using electromyographic (EMG) signals from residual muscles [4]. As the field of HMI in rehabilitation and medicine rapidly evolves, so do attempts to increase the ease of use and decrease the cognitive demand on the user. A recent example includes incorporating haptic feedback in robotic surgery designed to reduce cognitive overload [5].

In the field of prosthetics, the past decade has seen advances in human interaction for controlling prosthetic limbs with improved myoelectric control strategies [6], improved prosthesis design [7], and implantable electrodes for high-performance neural control of robotic limbs [8], [9]. Surgical techniques such as targeted muscle reinnervation (TMR), which allows transfer of residual limb nerves to alternative muscle sites for facilitation of EMG signals have been pioneered to enhance prosthesis control [10]. TMR has even been shown to enable the potential for somatosensory feedback in amputee patients [11], [12], [13], which may significantly reduce the attentional demands of using a prosthesis since users currently rely on visual feedback for tasks such as grasping [14]. All of these advances are intended to make prosthesis control as intuitive and functional as possible since basic activities of daily living, such as dressing, toileting, and ambulation can be very challenging for individuals with amputations. As such, calls for future research have included studies on cognitive workload in conjunction with development of new technologies to improve performance [15].

Researchers and clinicians in prosthetics have made recent efforts to review, improve, and validate outcome measures for prosthetic limb users [16], [17], however, most assessments currently used are qualitative, relying on subjective observations from clinicians or self-reports from subjects [18]. More broadly, in the field of HMI, some studies have employed self-report scales such as the NASA Task Load Index (NASA-TLX), however, such scales are also subjective [19]. Despite efforts to improve prosthetics and HMI by reducing the attentional burden, standardized methods for objectively quantifying cognitive workload in the field of HMI, rehabilitation, and prosthetics have not been established. More objective approaches to understanding cognitive strategies and workload in prosthetics and rehabilitation have included efforts to measure eye gaze [20], [21]. Although gaze behavior, blinking rate, and pupil dilation can reflect cognitive workload, they can also reflect other environmental and task factors such as ambient light [21], [22]. Such applications of eye tracking behavior remain promising, especially when used in conjunction with measures of cortical dynamics [23].

The electroencephalogram (EEG) offers the potential to examine cognitive effort with precise temporal resolution and freedom of movement during data collection, facilitating adaptability to clinical, operational, or real-world settings [24], [25], [26], [27]. Remarkably, although efforts to use measures of cortical dynamics such as EEG are increasingly abundant in the literature for control of assistive devices [28], [29], [30], [31], [32], [33], [34], EEG measures of cognitive workload for evaluation of new HMI technologies have not been adapted and applied to this field.

One EEG approach is based on the allocation of neural resources to a primary task, and the subsequent “attentional reserve” available for the processing of any additional demands [35]. Event-related potentials (ERPs) are EEG peaks averaged in the time domain and time-locked to discrete stimuli. ERPs have been used to examine cognitive workload during task performance based on the inverse relationship between cognitive workload of the primary task, and the amplitude of ERPs elicited by the secondary task or probe. When cognitive demand on the primary task is high, ERP amplitudes to a secondary stimulus are low, reflecting the reduced available neural resources allocated to process the distractor under high cognitive workload [30].

Early ERP studies employed dual-task paradigms, whereby the participants were engaged in a primary cognitive task while simultaneously attending to a secondary auditory or visual target-detection task [35], [36], [37], [38]. However, secondary discrimination tasks can alter the nature of the primary task [39], leading some researchers to adopt a strategy of using an irrelevant probe to elicit ERPs during primary task performance [40], [41], [42]. The irrelevant probe method is more conducive to preserving the ecological validity of the primary task, and more generalizable to naturalistic situations [43]. To maximize the saliency of the probe stimuli and optimize the attentional response, recent studies have used of rare novel sounds in place of common tones [42], [44]. Miller and colleagues [42] combined this approach with graded manipulation of task difficulty to examine cognitive workload while participants played the video game Tetris. The graded difficulty included three conditions: passively watching (view), playing at level 1 (easy), and playing at level 8 (hard). The authors found that four ERP components, including the N100, P200, P300, and late positive potential (LPP), significantly distinguished cognitive workload in the three conditions, all exhibiting an inverse relationship between amplitude and level of difficulty. The early ERP components are thought to reflect obligatory perceptual processing of the auditory stimulus (N100, P200), and later ERP components are thought to reflect cognitive evaluation of the stimulus [45], [46]. However, the early components have also been shown to be sensitive to attention and cognitive workload [41], [42], [44].

### Myoelectric Limb Control: Direct Control (DC) and Pattern Recognition Control (PRC)

Several advanced prosthetic limbs have recently reached the market [47] and several more are in development. The most promising approach to controlling such advanced devices is myoelectric control, or use of EMG signals from residual muscles. The conventional method of myoelectric control, called direct control (DC), uses the amplitude of the EMG signal from a single muscle to control a single movement. As such, DC requires a pair of agonist/antagonist muscles (e.g. biceps/triceps) to control a single degree of freedom (DOF), such as hand open/close. Control of two DOFs would require either four separate muscle sites for control (two sets of agonist/antagonist muscles), or a mechanism for switching between DOFs while using the same two muscles. A newer method of myoelectric control, called pattern recognition control (PRC) extracts features from the EMG signal associated with the intended movement of the patient, and passes them to a classifier, which outputs them as a movement class label (e.g. hand close). The algorithms can be trained for multiple DOFs (e.g. hand open/close, wrist extension/flexion, and wrist pronation/supination) enabling the patient to make movements in all three DOFs just by thinking about making the natural movement in the phantom limb. As such, researchers have speculated that PRC is more intuitive and lower in cognitive burden than DC. However, this has not been empirically tested using an objective measure of cognitive workload.

### Statement of Purpose

The purpose of this study was to establish the efficacy and potential of a new outcome measure for cognitive workload in prosthetics research, and to compare the relative cognitive workload of two different prosthetic control approaches using the new measure. EEG/ERPs have been shown to reflect cognitive workload in previous studies, while allowing the participant to execute ecologically valid tasks such as baggage screening [23] or video gaming [48], and were explored here for application to clinical studies examining prosthetic limb use. Healthy control participants engaged in a myoelectrically controlled virtual arm task under three conditions: simply watching the arm move (view), moving the hand in 1 DOF (easy), and moving the hand in 3 DOF (hard). Participants were trained and tested using DC, the most successful clinically available method, and a state-of-the-art PRC approach. ERPs were examined for differences in amplitude between the three levels of difficulty, and compared between DC and PRC conditions. Based on previous reports, we expected the amplitude of the ERPs to exhibit an inverse relationship to the level of difficulty of the virtual arm task, which would confirm the potential for this as a measure of cognitive workload in future prosthetic and rehabilitation technology research.

## Materials and Methods

### Participants

Twenty intact-limb individuals were recruited to participate in the study. All participants were inexperienced in using myoelectric control strategies. The sample size was determined based on a power analysis from previously published data [42]. Two participants were excluded from analysis following data collection due to excessive noise in the EEG signal, therefore, 18 participants (7M/11F) ranging in age from 21–38 years (mean  = 26.6) were included in the analysis.

### Ethics Statement

Written, informed consent was provided by all participants to participate in the study. The study was specifically approved by the Northwestern University institutional review board (IRB) (Ref no.: STU00062490).

### Procedures

Participants were trained and tested using both DC and PRC, with the order counter balanced. Participants made four visits to the Rehabilitation Institute of Chicago (RIC) over two weeks. The first visit was to train on one of the two myoelectric control strategies (DC or PRC), and the second visit during the same week was for testing during collection of EEG. The same procedure was followed the next week on the other myoelectric control strategy.

### Myoelectric Control of the Virtual Arm

**Figure 1:**
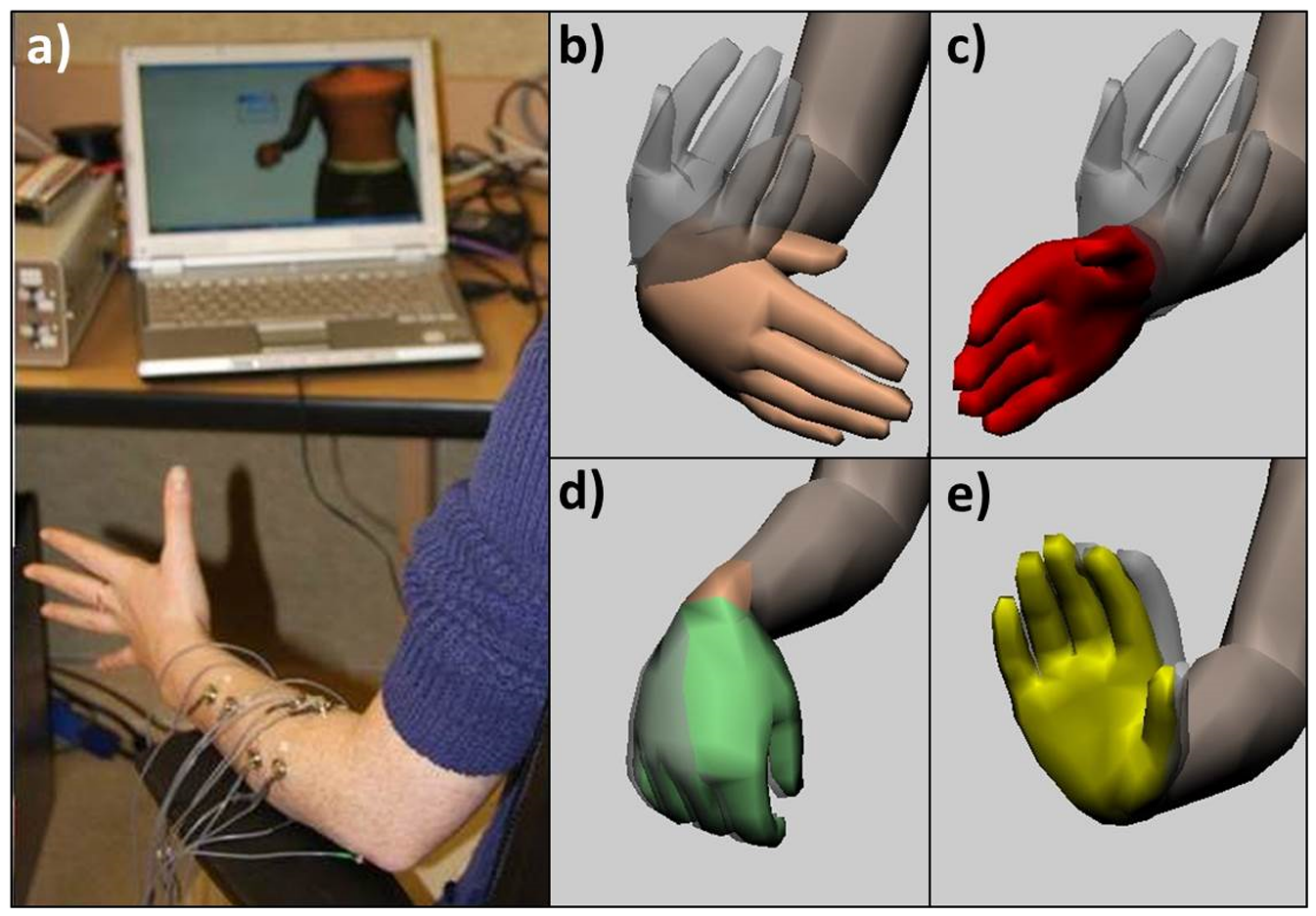
Electrode setup and virtual arm task in CAPS. a) Participant controlling the virtual arm using six bipolar electrodes (three visible in image) affixed to the non-dominant forearm. The small “go” text box above the virtual hand signals when the trial begins. b) The flesh-colored hand indicates the current hand position, and the gray hand indicates the target position. c) In the DC condition, a red hand flash indicates successful switching of the DOF through co-contraction of electrodes 1 and 2. d) Successfully acquiring the hand position results in the hand turning green and the end of the trial. e) If the target position is not successfully acquired within 24 sec, the hand turns yellow and a new trial begins.

Myoelectric control of the virtual arm was accomplished using a software suite developed at the RIC called Control Algorithms for Prosthetic Systems (CAPS). CAPS was designed for clinical testing and training of patients using myoelectric inputs to control real-time avatar motion with both DC and PRC algorithms, and supports communication with external programs. The use of CAPS for target acquisition testing in 3 DOF has been previously reported in patients with transradial amputation [49], and was used in the current paper. EMG inputs for virtual arm control were acquired using six bipolar EMG electrodes placed in predetermined positions on the forearm (Figure 1a). Only Channels 1 and 2 were used to control the virtual arm in the DC condition, and all six channels were used in the PRC condition. Electrodes 1 and 2 were placed to optimize amplitude acquired during wrist flexion and extension, respectively. Channels 3 and 4 were placed equidistant to channels 1 and 2 on the medial and lateral sides of the forearm. Channels 5 and 6 were placed distally on the dorsal and ventral aspects of the wrist. Signals were amplified and high-pass filtered at 20 Hz, and data were sampled at 1 kHz by an analog-to digital converter (USB-1616FS; Measurement Computing Corp, Norton, Massachusetts). For the DC condition, gains were set to optimize control of the hand, and to optimize switching between DOFs. Switching was accomplished in DC using a co-contraction of the flexor and extensor muscles by making a fist and relaxing in quick succession. In the PRC condition, participants performed movements to train the algorithms for the appropriate DOFs.

#### Training

All six electrodes were applied to the non-dominant arm during training in both conditions for consistency. After the electrodes were applied, the signals were viewed and checked for quality using a signal viewing tool within CAPS. Participants then performed 20 trials in each single DOF (wrist extension/flexion, wrist pronation, supination, hand open/close) for a total of 60 trials to practice for the easy condition. Participants then completed a total of 40 trials in the hard condition, which required moving the virtual arm in all 3 DOFs to complete the trial (Figure 1).

#### Testing

The virtual limb was presented on a 17″ monitor connected to a computer that ran the CAPS software integrated with Matlab for presentation of sounds and associated EEG triggers. Testing consisted of three conditions: 1) passively viewing the virtual hand execute the 3DOF condition; 2) performing the 1DOF task for 24 trials in each of the three degrees of freedom (easy); 3) performing 32 trials of the the 3DOF task (hard). Participants received the view condition first 50% of the time (view, easy, hard), and last 50% of the time to counterbalance the novelty of the sounds across conditions (easy, hard, view); participants always completed the easy task prior to the hard task. Trials consisted of a “+” in the center of the screen as a visual prompt prior to start of the trial, followed by presentation of the task cue, consisting of the current position of the hand in gray, superimposed over the flesh colored target position of the hand. Participants had four seconds to plan the movement prior to receiving a “Go” stimulus to start moving. Moving prior to the “Go” stimulus would result in no movement of the virtual hand. Successfully moving the virtual hand over the target position resulted in the hand turning green and completion of the trial. If they did not successfully reach the target position within 24 seconds, the trial ended and the hand turned yellow before beginning the next trial. Participants received auditory probes at random intervals during the trials (0–3 probes per trial) (Figure 1). The novel distractor sounds were the same as those used in Miller et al., (2011), which were selected from Fabiani et al., (1996) [50].

#### EEG

Continuous EEG was recorded during testing using a lycra cap, (Electro-Cap International Inc.). Data were acquired from 16 sites adapted from the 10–20 system [51] and referenced to the left earlobe (A1). Eye channels were place above and below the left eye, and on the outer canthi of both eyes to record eyeblinks. Impedences were kept below 10 KΩ, and channels were amplified 1000 times using Neuroscan Synamps^2^ with a sampling rate of 1000 Hz. Online bandpass filters were set from .01–100 Hz. ERPs were obtained by extracting epochs from 100 ms pre-stimulus onset to 10 00 ms post-stimulus onset, baseline correcting on the pre-stimulus interval, and bandpass filtering from 1–15 Hz. Each epoch was visually inspected for artifact, and trials with excessive movement or blinks were deleted (“trials” in the ERP analysis refers to the number of auditory probes, and subsequent single-trial ERPs, not the number of task trials). At least 30 trials in each condition were used for averaging; in the event that 30 clean trials were not available for each condition, the participant was excluded from analysis. The mean amplitude for each ERP component at sites Fz, Cz, and Pz (frontal, central, and parietal midline sites, respectively) was calculated using the method reported by Handy [52], and used in Miller et al., [42]. Narrow time windows for each peak were centered around the grand average peak, and average amplitude was calculated in the following time windows: N100 = 105–120 ms; P200 = 190–205 ms; P300 = 295–330 ms; LPP = 570–590 ms.

#### Performance

The target acquisition testing in CAPS generates log files during testing for performance analysis. The percentage of trials successfully completed in each condition, and time to complete successful trials (seconds) was examined.

#### Self-report

Immediately after completion of testing in the DC condition and the PRC condition participants filled out a self-report questionnaire indicating the perceived difficulty of controlling the arm. The questions were taken from questionnaires commonly administered by clinicians at the RIC (Appendix Survey S1). It consisted of 7 questions on a five-point Likert scale, with questions 1, 3, and 7 reverse scored such that a high score reflected a high level of perceived difficulty.

#### Statistical Design

Our primary hypothesis was that the ERPs would reveal differences in cognitive workload between the view, easy, and hard conditions, and that those ERPs that reflected cognitive workload as a main effect would also distinguish PRC and DC during the hard condition. To assess the efficacy of the ERPs for delineating cognitive workload, main effects on the cognitive workload factor were analyzed using 3 (view, easy, hard) ×2 (DC, PRC) repeated measures ANOVAs. Separate ANOVAs were conducted on average amplitude for each peak reported to reflect cognitive workload previously using this paradigm [Miller et al., [42]]: N1 (Cz), P2 (Fz, Cz, Pz), P3 (Pz), and LPP (Pz). To compare differences between myoelectric control conditions, two-tailed paired-samples t-tests were conducted in the easy and hard conditions on peaks that exhibited main effects for cognitive workload in both the current analysis, and in Miller et al. Performance results (% correct, time to complete trials) were compared between DC and PRC in each condition (easy, hard) using two-tailed paired-samples t-tests. Self-report was compared between DC and PRC using a two-tailed paired samples t-test. Simple correlations were also examined between ERPs exhibiting cognitive workload effects and performance, ERPs and self-report, and self-report and performance.

## Results

### EEG analysis

**Figure 2:**
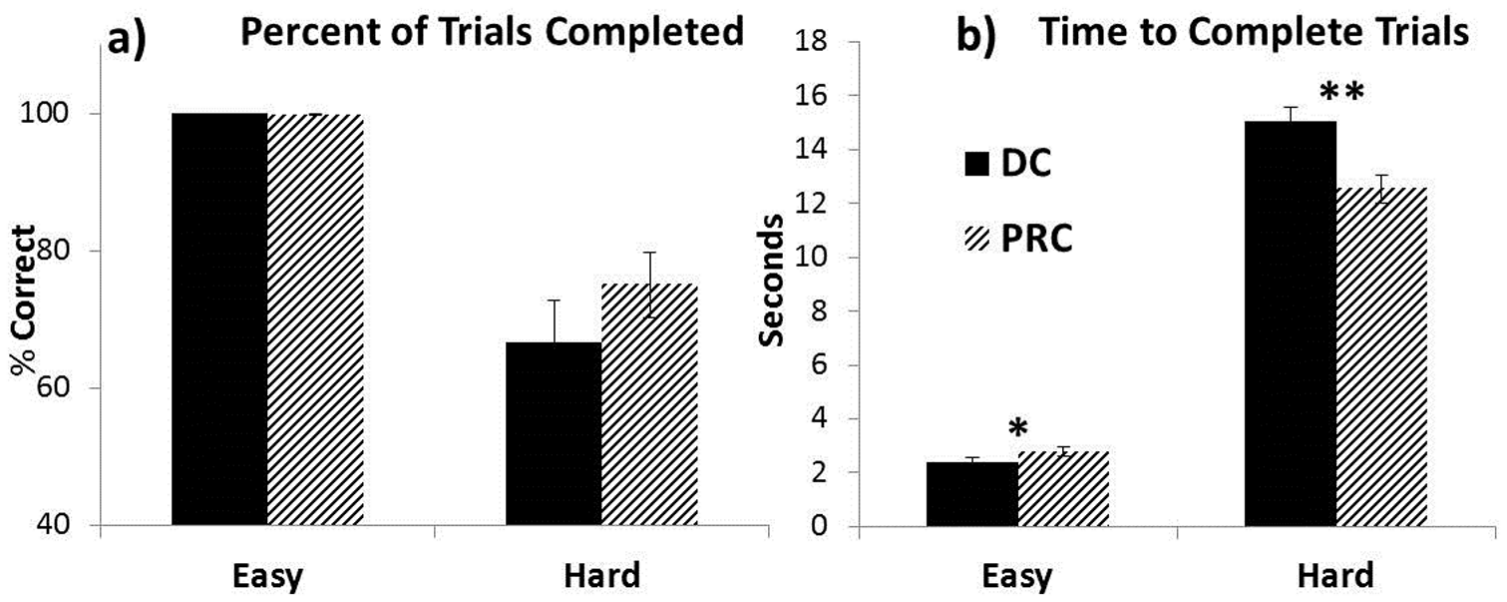
Virtual arm task performance. a) Percentage of trials completed within 24 seconds. b) Average time to complete successful trials; participants performed significantly faster in the easy condition using DC, and significantly faster in the hard condition using PRC (*p<0.05; **p<0.01.).

Figure 2 illustrates the strong general inverse relationship between ERP amplitudes and cognitive workload for P200, P300, and LPP. Main effects and ERPs are pictured for electrodes Cz and Pz, where most of the effects occurred. No significant main effects or interactions emerged for cognitive workload on the N1 component. Consistent with Miller et al. (2011) [42], the main effects for cognitive workload were significant at all three electrode sites (Fz, Cz, Pz) for P200: Fz (F_2,34_ = 5.15; p = 0.011), Cz (F_2,34_ = 12.90; p<0.001), and Pz (F_2,34_ = 5.03; p = 0.012). P200 effects at Cz and Pz are pictured in Figure 2 (a–d). Post-hoc tests revealed that for P200 at Cz, the view condition differed from both the easy and hard conditions. For P200 at Fz and Pz, the hard conditions differed from the view conditions. Significant main effects emerged on P300 at Pz (F_2,34_ = 7.67; p = 0.002) and LPP at Pz (F_2,34_ = 5.61; p = 0.008), and are pictured in Figure 2 (b, e, f). For both P300 and LPP, pots-hoc testing revealed that the hard conditions were significantly different from the view conditions.

**Figure 3:**
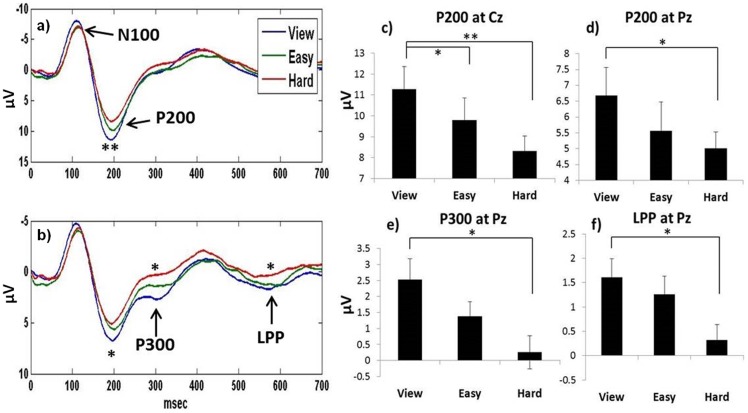
ERPs and main effects for the cognitive workload measure on the view, easy, and hard conditions for DC and PRC combined. Note that positive and negative on the y-axis are traditionally reversed for ERP graphs; as such positive is graphed down. a) Electrode Cz, where the P200 effect was most prominent. b) Electrode Pz, where the P200, P300, and LPP all exhibited cognitive workload effects. c–f) Average amplitude graphs for P200 at Cz and Pz, P300, and LPP (*p<0.05, **p<0.01.).

To compare DC and PRC, two-tailed paired samples t-tests were run on hard conditions for P200 (Fz, Cz, Pz), P300 (Pz), and LPP (Pz), which all showed main effects for cognitive workload. Because t-tests do not control for multiple comparisons like Tukey HSD, the number of analyses was limited to only the peaks that exhibited significant main effects in the current study, and were also reported to be significant in Miller et al. [42]. A significant difference in average amplitude in the hard condition emerged for LPP (t_17_ = −2.35, p = 0.031), with PRC exhibiting a higher LPP amplitude relative to DC. Figure 3 illustrates the ERPs at Pz for DC and PRC in the hard condition.

### Performance

**Figure 4:**
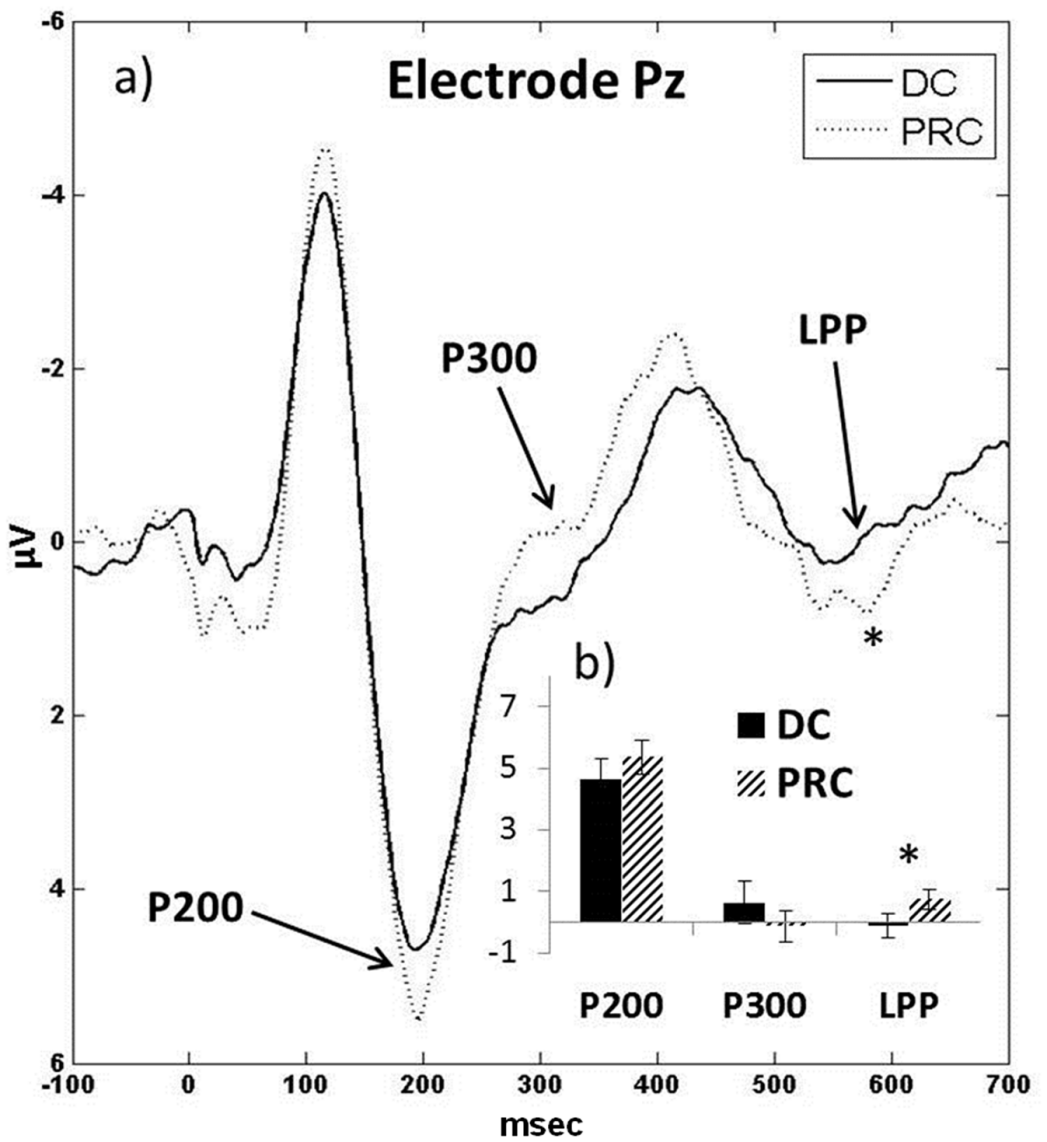
DC and PRC ERPs and amplitudes for electrode Pz in the hard condition. a) Visual inspection of the ERP shows that in the hard condition the P300 was not visually prominent, and close to zero. b) Although LPP was not visually prominent, the difference was significant between DC and PRC, with higher amplitude for PRC (*p<0.05.).

Figure 4 illustrates the results for trials completed (4a), and time to complete trials (4b). No significant performance differences for percent correct emerged between DC and PRC in either the easy or hard conditions. Mean completion time was significantly lower for DC (2.4 sec, SD = 0.81) relative to PRC (2.8 sec, SD = 0.81) in the easy condition (t_17_ = −2.34, p = 0.031), however, it was significantly higher for DC (15.1 sec, SD = 2.18) relative to PRC (12.5 sec, SD = 2.19) in the hard condition (t_17_ = 3.66, p = 0.002). The results indicate that DC may be more effective relative to PRC for simple (1 DOF) tasks, but more challenging for complex (3 DOF) tasks.

### Self-report

Self-report was not obtained from one participant, so the analyses reflect scores from 17 participants. There were no differences between DC (mean  = 15.1, SD = 3.8) and PRC (mean  = 16.3, SD = 4.4) on the self-report questions examining difficulty.

### Correlations

ERPs exhibiting a cognitive workload effect (P200, P300, LPP) did not correlate significantly with performance in either the DC condition or the PRC condition. This suggests that cortical effort during the tasks was independent of performance. Self-report did correlate significantly with mean completion time in the hard condition for both the DC (r = 0.70, p = 0.002) and PRC (r = 0.64, p = 0.006), however, it did not correlate with ERPs in either the DC or PRC conditions. This result suggests that participants self-rated the difficulty consistent with their performance, and not consistent with the cortical effort required for task completion.

## Discussion

EEG/ERPs have been explored as measures of cognitive workload during performance of real-world, ecologically valid tasks ranging from flight simulation [37], to video games [42], [48], to baggage screening [23], and even soldiers in operational settings [25]. The current study adapted one such approach to examine the use of ERPs as a cognitive workload outcome measure for HMI, specifically, during myoelectric prosthetic limb control. Consistent with previous research, the results indicated an inverse relationship between ERP amplitude and task difficulty, supporting the efficacy of this measure.

### ERPs and Cognitive Workload

Significant main effects of cognitive workload were demonstrated in the omnibus tests for P200, P300, and for LPP. The results are remarkably consistent with the results of Miller et al., [42], who employed the same paradigm to examine cognitive workload during play of the video game Tetris. Consistent with the previous report, P200 exhibited cognitive workload effects across all three sites (Fz, Cz, Pz), with the strongest effect over the vertex (Cz). Main effects were also found for P300 and LPP at electrode Pz. No significant cognitive workload effects were found in the current study for N100. Although the N100 component is believed to reflect early sensory auditory processing, it has been shown to be sensitive to attention in previous studies [41], [42], [44].

The P300 is one of the most commonly studied cognitive ERP components over the last several decades [for a review, see [53]]. In the current study the P300 exhibited a strong cognitive workload effect, however, in the hard condition for both DC and PRC, the P300 was virtually absent in the grand average across all subjects. The P200 and LPP components have received less examination in cognitive neuroscience in general, and in the cognitive workload literature relative to P300, yet both P200 and LPP exhibited strong effects in the current paper, and a previous paper using a very similar paradigm [42], and identical novel auditory tones [50]. Although the P200 was considered by some researchers in early studies to be the tail end of the N100-P200 complex, more recent studies have demonstrated that the P200 is an independent component that can be elicited through visual, somatosensory, and auditory modalities, and is maximal over the vertex [for a review see [54]]. It has been suggested to represent early processing of emotionally or motivationally relevant stimuli [55]. The LPP, often referred to as the late positive component or complex (LPC), has been proposed to reflect continued or enhanced elaborate processing of emotional or arousing stimuli [55], [56], and has been suggested to exhibit positivity with latencies ranging from 300 milliseconds to several seconds [57]. In the current study, the grand average across all subjects was used to identify the LLP peak in a relatively narrow time window for analysis [52], from 570–590 milliseconds, which is temporally consistent with the LLP reported in Miller et al., [42].

### Direct Control vs Pattern Recognition

The second goal of the present study was to compare DC and PRC using the ERP measures. Because the PRC method allows the user to make natural movements (in the case of non-amputee controls) or imagine natural movements (in amputee patients) to accomplish the tasks, PRC has been speculated to be more intuitive and lower in cognitive demand than DC, although this notion had not previously been examined empirically.

Having exhibited significant cognitive workload effects across both DC and PRC conditions, P200, P300, and LPP were compared between DC and PRC conditions in the hard and easy tasks. Only LPP exhibited a significant difference between DC and PRC, and only in the hard condition. Amplitude was higher in the PRC condition than the DC condition, consistent with lower cognitive workload for a complex task using PRC relative to DC. This is interpreted cautiously however, since the P200 and P300 components were not different between DC and PRC.

Although PRC has been speculated to be lower in cognitive workload relative to DC in the absence of previous empirical evidence, many aspects of the cognitive workload required to complete the tasks are inherent to both DC and PRC, and were held constant in this study. One of the more challenging aspects of the task was the mental visual rotation necessary to see the current hand position, and decipher the hand movements in 3 DOFs required to achieve the target position. This aspect of the cognitive burden was consistent between DC and PRC, and was very challenging for the participants. Beyond deciphering how the virtual hand needed to move, the participants then had to complete the appropriate sequence of contractions to accomplish the proper movement. In DC, this required frequent switching between DOFs, and the mental conversion of remembering how to use movements in 1 DOF (wrist extension/flexion) to control 3 DOFs (e.g. extending the wrist to supinate the wrist and open the hand, and flexing the wrist pronate and close the hand). Although the pattern of contractions for DC may be less intuitive, the participants learned fairly quickly. Yet, despite subtle differences in the cognitive burden between DC and PRC on the hard task in this study, the significant difference in LPP amplitude indicates the sensitivity of ERP studies for detecting subtle differences in cognitive workload.

### Self-report

One of the most important features of EEG as an outcome measure is objectivity. Researchers in the prosthetics field currently rely on observation by trained clinicians, and self-report from patients or family members to evaluate the mental workload of using a prosthesis, both of which are subjective and prone to bias. The self-report questions administered here correlated with task performance in the hard condition for DC and PRC, but did not correlate with the ERP measures of cognitive effort. This suggests that the participants' perceived effort reflected knowledge of their own performance rather than the actual cortical resources required for the task. In retrospect, this was likely influenced by the sequence of the procedures and the immediate feedback they received. The questionnaire was administered immediately after seeing the performance results for the hard task at the end of testing. As such, knowledge of their performance was fresh in their mind as the participants completed the questionnaire. Although future studies can be designed to administer brief self-reports more frequently, or prior to such salient feedback on the task, the correlation of the self-report with performance rather than cortical activation during task execution in this study illustrates the subjectivity of self-report, and the need for an objective outcome measure of cognitive workload to supplement self-reports in the rehabilitation and prosthetics field. Such a measure will be informative for evaluating current technology, and guiding efforts for new technology.

### Advantages of EEG/ERPs

This study represents the first attempt to quantify cognitive workload in myoelectric limb control, and the results are promising. Three separate ERP components exhibited significant cognitive workload effects illustrating the inverse relationship between ERP amplitude to the novel sounds and task difficulty. The paradigm is easily adaptable to research on a variety of HMI tasks where cognitive workload is relevant, including control of exoskeletons or surgical robots. Wireless EEG caps can enable real-time and offline EEG analysis for ecologically valid movements and ambulatory tasks. By examining the cortical resources available during task engagement for processing of additional stimuli, the paradigm is agnostic to strategy, and allows objective examination of cognitive workload when strategy and cortical activation patterns for two tasks may be different, as in the two myoelectric prosthesis control strategies examined here. Other approaches to measuring cognitive workload with EEG, such as pattern recognition and neural networks [26], [58], [59], [60], are optimal for monitoring workload for a specific task in an individual, but may introduce bias when comparing cognitive workload between two similar, but different tasks. However, more sophisticated signal processing approaches may increase the sensitivity of the ERP approach used in this study.

An additional advantage of the approach presented here is the sensitivity of the information acquired through a small number of electrodes. Although 16 channels of EEG data were obtained in this study, the results could have been obtained using only three or fewer midline electrodes (Fz, Cz, Pz), a few eye channels, a ground, and a reference. Newer EEG caps are being designed to be donned and doffed with ease, and without gel. Furthermore, other EEG measures where additional electrodes are employed can simultaneously address task difficulty, regional activation, and functional communication between different cortical regions to examine sensory, motor, and cognitive demands of a task such as prosthesis use [61], [62].

### Limitations and Future Studies

The current study was limited to healthy participants with in-tact limbs, and conducted using a virtual environment. As such, future efforts will extend to upper and lower limb amputee patients, and should be adapted to activity of daily living tasks such as object manipulation and stair climbing. The advantage of the attentional reserve paradigm of assessing cognitive workload is the broad adaptability of the approach for comparison of different tasks and strategies in a range of HMI environments.

Although the results in this study pertain only to healthy control participants, and the specific virtual task in this study, the ERP approach as an outcome measure is a promising technique to evaluate new emerging prosthetic technologies and clinical approaches. For instance, the PRC method tested here required making one DOF movement at a time. In other words, the hand could not be supinated and closed simultaneously. However, studies are underway to adapt PRC measures for multiple simultaneous movements [63], which may continue to improve efficiency and decrease cognitive demand. Other ongoing research efforts address the lack of sensory feedback from a prosthesis, requiring a prosthesis user to rely solely on visual information to control tasks such as grasping. Studies exploring tactile and other forms of feedback seek to enhance performance while reducing the attentional demands [11], [12], [13], [14]. Even surgical techniques such as targeted muscle reinnervation [TMR; [10]], designed to optimize the EMG signal in the residual muscle, have been developed with the goal of making prosthetic limb control more natural and intuitive. The ERP method described here can be adapted to evaluate cognitive workload with these or other emerging rehabilitation technologies.

## Conclusions

The goal of this study was to examine the efficacy of using ERPs as an outcome measure for cognitive workload in HMI, specifically, during myoelectric prosthesis control. Secondly, to use the ERP measures exhibiting main effects for cognitive workload to compare two myoelectric strategies, DC and PRC. The results indicated an inverse relationship between cognitive workload and amplitude on P200, P300, and LPP following presentation of novel auditory probes. LPP amplitude was higher on the complex task using PRC compared to DC, suggesting a subtle difference in cognitive workload between DC and PRC.

The current study examined only a virtual arm task in healthy participants with in-tact limbs, and requires replication in patients with amputations, and adaptation to manipulation and mobility tasks using a prosthesis. However, the ERP approach, and other EEG measures are adaptable to a variety of HMI tasks as objective outcome measures of cortical and attentional effort.

## Supporting Information

Appendix Survey S1 Virtual/Prosthetic Arm Control Survey.
(DOCX)
