# Supplementary material for: A Simple ERP Method for Quantitative Analysis of Cognitive Workload in Myoelectric Prosthesis Control and Human-Machine Interaction
Source: PLoS One. 2014 Nov 17;9(11):e112091. doi: 10.1371/journal.pone.0112091 (PMC4234315; doi:10.1371/journal.pone.0112091)
Supplement: Appendix Survey S1 — Virtual/Prosthetic Arm Control Survey. (DOCX) [file pone.0112091.s001.docx]

Appendix A

**Virtual/Prosthetic Arm Control Survey**

**Administrative use:** Participant identification code __________ Condition_____ Date____________

**Instructions:** Please complete the following survey based on your experience with the virtual/prosthetic arm.

Please answer the following questions**:**

**1. The virtual/prosthetic arm responded as I expected.**

Strongly Disagree 1 2 3 4 5 Strongly Agree N/A

**2. It was hard to plan my movements.**

Strongly Disagree 1 2 3 4 5 Strongly Agree N/A

**3. It was easy to make the virtual/prosthetic arm move when I wanted.**

Strongly Disagree 1 2 3 4 5 Strongly Agree N/A

**4. I felt fatigued after using the arm.**

Strongly Disagree 1 2 3 4 5 Strongly Agree N/A

**5. There were a lot of unintended movements of the virtual/prosthetic arm.**

Strongly Disagree 1 2 3 4 5 Strongly Agree N/A

**6. I was frustrated using the virtual/prosthetic arm.**

Strongly Disagree 1 2 3 4 5 Strongly Agree N/A

**7. The virtual/prosthetic arm moved at a desirable speed.**

Strongly Disagree 1 2 3 4 5 Strongly Agree N/A

**Additional comments:**
